# Oviposition deterrent activity from the ethanolic extract of **Pongamia pinnata, Coleus forskohlii**, and **Datura stramonium** leaves against *Aedes aegypti* and **Culex quinquefaciatus**

**DOI:** 10.4103/0973-1296.71796

**Published:** 2010

**Authors:** S. Swathi, G. Murugananthan, S. K. Ghosh

**Affiliations:** *Department of Pharmacognosy, PES College of Pharmacy, 50 Feet Road, Hanumanth Nagar, Bangalore - 560 050, India*; 1 *National Institute of Malaria Research, ICMR, Near Trumpet Bus Stop, Bangalore - 561 102, India*

**Keywords:** *Aedes aegypti*, *Coleus forskohlii*, Culex quinquefasciatus, Datura stramonium, oviposition deterrent, *Pongamia pinnata*

## Abstract

Mosquitoes are responsible for spread of many diseases than any other group of arthropods. Diseases such as malaria, filariasis, dengue hemorrhagic fever (DHF), and chikunguinya are real threat to mankind. In the present study, ethanolic extracts of leaves of **Pongamia pinnata, Coleus forskohlii**, and **Datura stramonium** were evaluated for oviposition deterrent activity against *Aedes aegypti* and **Culex quinquefasciatus**. The oviposition deterrent tests of ethanolic extract of **Pongamia pinnata, Coleus forskohlii**, and **Datura stramonium** leaves reduced egg laying by 97.62%, 77.3%, 100% against *Aedes aegypti* and 59.10%, 39.22%, 82% against **Culex quinquefasciatus** at higher concentration (0.1%).

## INTRODUCTION

Mosquitoes are the vectors for the dreadful diseases of mankind. Of all the insects that transmit diseases, mosquitoes represent the greatest menace.[1] While most people consider mosquitoes as an annoyance, these tiny assassins have the potential and lethal capacity to kill more than a million victims a year around the world.[2] Prevalence of mosquito-borne diseases is one of the world’s most health hazardous problems.[3] One of the methods available for the control of mosquitoes is the use of insecticides. Chemical control using synthetic insecticides had been favorable so far because of their speedy action and easy application.[1] Synthetic insecticides are toxic and adversely affect the environment by contaminating soil, water, and air. Botanical pesticides are promising in that they are effective, environment-friendly, easily biodegradable, and also inexpensive.[4]

The mosquito *Aedes aegypti* acts as a vector for an arbovirus responsible for yellow fever in Central and South America and in West Africa. It is also the vector of dengue hemorrhagic fever, which is endemic to South East Asia, the Pacific islands area, Africa.[5] **Culex quinquefasciatus** Say is the main vector of bancroftian filariasis. Global prevalence of lymphatic filariasis is 120 million and population at risk is 1.3 billion. In India, there may be up to 31 million microfilareamics and 23 million cases of symptomatic filariasis.[6] Urbanization and changed lifestyles mainly contribute to the proliferation of larval habitats resulting in disease epidemics.[7]

It is estimated that every year at least 500 million people in the world suffer from one or the other tropical diseases that include malaria, lymphatic filariasis, schistosomiasis, dengue, trypanosomiasis, and leishmaniasis. Of late chikungunya, a serious mosquito borne epidemic has gained momentum in India. These diseases not only cause high levels of morbidity and mortality, but also inflict great economic loss and social disruption on developing countries such as India, China, etc.

Mosquito population can be reduced by disrupting its oviposition.[7] To avoid the propensity of bioaccumulation and induction of malignancy in nontarget animals, a safe and more congenial method of vector control by natural and cheaper means of using plants as insecticides became popular.[8] Plants are considered as a rich source of bioactive chemicals and they may be an alternative source of mosquito control agents.[9] The co-evolution of plants with insects has equipped them with a plethora of chemical defenses, which can be used against insects. Since botanicals are less likely to cause ecological damage, a large number of plants have been screened for their insecticidal activities against mosquitoes and some of these have been found to possess promising effects.[10]

The present study was an attempt to explore oviposition deterrent activity from ethanolic extract of **Pongamia pinnata, Coleus forskohlii**, and **Datura stramonium** leaves against *Aedes aegypti* and **Culex quinquefasciatus**.

## MATERIALS AND METHODS

### Collection of plants and extraction

Fully developed leaves of **Pongamia pinnata, Coleus forskohlii**, and **Datura stramonium** were collected and voucher specimens have been authenticated by Dr. Rajanna (Botanist), Department of botany, G.K.V.K, Bangalore, India. The leaves were washed with tap water, shade dried, and powdered. The powdered plant material was loaded in soxhlet apparatus and was extracted with ethanol. The solvent from the extract was subjected to vacuum evaporator to collect the crude extract. Standard stock solutions were prepared by dissolving the residues in the ethanol. These solutions were used for oviposition deterrent bioassay.

### Oviposition deterrent bioassay

The oviposition deterrent test was performed using the method of Xue *et al*.[11] against *Aedes aegypti, Anopheles stephens,* and **Culex quinquefaciatus**. Fifteen gravid female were (10-day-old, 4 days after blood feeding) transferred to each mosquito cage (45 * 38 * 38 cm) covered with a plastic screen, with a glass top, and a muslin sleeve for access. A 10% sucrose solution was available at all times. Serial dilutions of leaf extract were made in ethanol. Enamel bowls containing 100 ml of rainwater were treated with leaf extract to obtain test solutions of 0.01, 0.025, 0.05, 0.075, and 0.1%. Two enamel bowls holding 100 ml of rainwater were placed in opposite corners of each cage, one treated with the test material, and the other with a solvent control that contained 1% ethanol. The positions of the bowls were alternated between the different replicates so as to nullify any effect of position on oviposition. Three replicates for each concentration were run, with cages placed side by side for each bioassay. All experiments were run at ambient temperature (27 ± 2°C) with relative humidity of 70–80%. After 24 h, the number of eggs laid in treated and control bowls was recorded.

The percent effective repellency for each leaf extract concentration was calculated using the following formula.[12]

$$\mathrm{ER}\%\;=\;\frac{\mathrm{NC}\;-\;\mathrm{NT}\;*100}{\mathrm{NC}}$$

Where,

ER = Percent effective repellency

NC = Number of eggs in control

NT = Number of eggs in treatment

## RESULTS

Results of the oviposition deterrent activity of **Pongamia pinnata, Coleus forskohlii**, and **Datura stramonium** ethanolic leaves extract against *Aedes aegypti* and **Culex quinquefasciatus** are presented in Table 1. The data was recorded and statistical data was calculated and presented.

**Table 1:** Oviposition deterrent activity of **Pongamia pinnata, Coleus forskohlii**, and **Datura stramonium** against gravid female *Aedes aegypti* and **Culex quinquefasciatus**

Conc (%)	Effective repellency (%)					
	*Pongamia pinnata*		*Coleus forskohli*		Datura stramonium	
	*Aedes aegypti*	Culex quinquefasciatus	*Aedes aegypti*	Culex quinquefasciatus	*Aedes Aegypti*	Culex quinquefasciatus
0.1	97.62	59.10	77.3	39.22	100	82.00
0.05	88.32	40.66	63.15	22.27	100	60.50
0.01	82.46	35.24	42.54	19.90	100	47.05
0.005	74.42	29.35	24.03	3.93	100	25.11

Conc - Concentration

## DISCUSSION

The laboratory oviposition deterrent tests of *Solanum trilobatum* acetone extract of leaves reduced egg laying at higher concentration (0.1%) by 90 – 99% against *Aedes albopictus* and 99.4% against *Anopheles* stephensi.[13] The present oviposition deterrent tests of ethanolic extract of **Pongamia pinnata, Coleus forskohlii**, and **Datura stramonium** leaves reduced egg laying by 97.62%, 77.3%, and 100% against *Aedes aegypti* and 59.10%, 39.22%, 82% against **Culex quinquefasciatus** at a higher concentration (0.1%).

From above results, we can conclude that **Datura stramonium** has more efficient oviposition deterrence against *Aedes aegypti* and **Culex quinquefasciatus** when compared to *Pongamia pinnata* and *Coleus forskohlii*.
